# ACKNOWLEDGEMENTS TO REVIEWERS

**DOI:** 10.2174/1573403X2106250901154609

**Published:** 2025-09-01

Bentham Science Publishers would like to thank and appreciate the co-operation from all reviewers for their constructive comments and feedback on the manuscripts submitted to **“Current Cardiology Reviews”**. Their efforts have contributed greatly to the high quality and continuous growth of the journal. Given below is the list of reviewers who reviewed articles for the Journal during 2024-2025:

Mohammad Abdelghani (Egypt)

Ahmed S. Abuzaid (United States)

Yong-Joo Ahn (Korea, South)

Alexander A. Aksenov (United States)

Antonio F. Amico (Italy)

Zhao An (China)

Ryuichiro Anan (Japan)

Aleksey S. Averin (Russia)

Juan J. Badimon (United States)

Federico J. Benetti (Argentina)

Ozlem Bilen (United States)

Erik A. Blackwood (United States)

Alexander Bobik (Australia)

Matthew J. Bock (United States)

Mark L. Brown (United States)

Pedro Bullon (Spain)

Ugur Canpolat (Turkiye)

Zhanqi Cao (China)

Arturo Cesaro (Italy)

Bing Chen (China)

Yan Chen (China)

Juyi Chen (Taiwan)

Tongshuai Chen (China)

Wen-Pin Chen (Taiwan)

Chungting Chen (Taiwan)

Qizhi Chen (China)

Jun Cheng (China)

Binghai Cong (China)

Alberto Cresti (Italy)

Guanglin Cui (China)

Yan Dai (China)

David W. DeGroot (United States)

Giovanni D. Di Salvo (Italy)

Ana C.O. Dos Santos (Brazil)

Paulo M.M. Dourado (Brazil)

Nevzat Erdil (Turkiye)

Ozlem Esen (Turkiye)

Zolkapli Eshak (Malaysia)

Soghra Farzipour (Iran)

Jian Feng (China)

Claudio Q. Fortes (Brazil)

Kentaro Fukuda (Japan)

Junying Gao (China)

Ying Gao (China)

Evgeny V. Grigoriev (Russia)

Hasan A. Gumrukcuoglu (Turkiye)

Umesh C. Gupta (Canada)

Mark C.P. Haigney (United States)

Masaki Hamamoto (Japan)

Yasmin S. Hamirani (United States)

Mert I. Hayiroglu (Turkiye)

Mert I. Hayiroglu (Turkiye)

Lei Hou (China)

Minghsiung Hsieh (Taiwan)

Zeping Hu (China)

Wei Huang (China)

Md S. Hussain (India)

Emrah Ipek (Turkiye)

Toshihiko Ishimitsu (Japan)

So-Ick Jang (Korea, South)

Mohammad H. Javanbakht (Iran)

Kouji Kajinami (Japan)

Alexandros N. Karavas (United States)

Shahzad Khan (Saudi Arabia)

Safir U. Khan (China)

Habib Khan (Canada)

James D. Kingsley (United States)

Kaan Kirali (Turkiye)

Kazufumi Kitagaki (Japan)

Melvin E. Klegerman (United States)

Lloyd W. Klein (United States)

Jae-Hong Ko (Korea, South)

Maria I. Kontaridis (United States)

Gerhard Kostner (Austria)

Richard Kovacs (United States)

Martin Landsberger (Germany)

Seung W. Lee (Korea, South)

Charles A. Lefkowitz (Canada)

Chiara Lestuzzi (Italy)

Jingdong Li (China)

Chuanbao Li (China)

Qing Li (China)

Shengli Li (China)

Shaoxiong Li (China)

Chao Liu (China)

Hailei Liu (China)

Xiwang Liu (China)

Haibo Liu (China)

Vernon J. Louw (South Africa)

Hao Lu (China)

Haiping Ma (China)

Stella M. Macin (Argentina)

Nani Maharani (Indonesia)

Fatemeh Mansouri (Iran)

Dante E. Manyari (Canada)

Eduardo Martinez-Abundis (Mexico)

Leonid N. Maslov (Russia)

Farzad Masoudkabir (Iran)

Dragan M. Matic (Serbia)

Yae Matsuo (Japan)

Sidharth Mehan (India)

Maryam Mehrpooya (Iran)

Mritunjay K. Mishra (India)

Olena I. Mitchenko (Ukraine)

Luis F. Montano (Mexico)

Yasushi Mukai (Japan)

Mark A.J. Newman (Australia)

Thach Nguyen (United States)

Masaaki Okutsu (Japan)

Rosamaria Oliart-Ros (Mexico)

Xianhong Ou (China)

Beatrice Paradiso (Italy)

Tilak K.R. Pasala (United States)

Hatice Pasaog Lu (Turkiye)

Kartik G. Patel (India)

Roberto C. Pedrosa (Brazil)

Nestor G. Perez (Argentina)

Louis P. Perrault (Canada)

Gianfranco Pintus (Italy)

Lyudmila M. Pivina (Kazakhstan)

Ekaterina A. Pravkina (Russia)

Weihua Qiao (China)

Yanwen Qin (China)

Yongwen Qin (China)

Henry C. Quevedo (United States)

Vladimir Rafalskiy (Russia)

Balasubramanian Rajkapoor (India)

Hugo R. Ramos (Argentina)

Umut Safer (Turkiye)

Helio C. Salgado (Brazil)

Gaetano Santulli (United States)

Jean-Francois Sarrazin (Canada)

Ferena Sayar (Iran)

Finney D. Shadrach (India)

Hongli Shan (China)

Azeem S. Sheikh (United Kingdom)

Changsheng Sheng (China)

Daoyuan Si (China)

Ieda de F.O. Silva (Brazil)

Zdenek Slavik (United Kingdom)

Andrea Sonaglioni (Italy)

Jinfang Song (China)

Gerald Soslau (United States)

Zhenxing Sun (China)

Zhenzhu Sun (China)

Paramjit S. Tappia (Canada)

Punniyakoti V. Thanikachalam (India)

Joho Tokumine (Japan)

Munesh Tomar (India)

Amit K. Tripathi (India)

Tetsuo Tsukamoto (Japan)

Sanae Tsumura (Japan)

Belma Turan (Turkiye)

Huseyin Uyarel (Turkiye)

Pradeep Vaideeswar (India)

Tao Wang (China)

Cheng Wang (China)

Dian Wang (China)

Lei Wang (China)

Gang Wang (China)

Mistiaen Wilhelm (Belgium)

Juefei Wu (China)

Jiaxin Xie (China)

Chennian Xu (China)

Xianxiang Xu (China)

Zhenyan Xu (China)

Yuejuan Xu (China)

Qian Xu (China)

Jing Xu (China)

Zhe Yang (China)

Mingchun Yang (Taiwan)

Xiaoyun Yang (China)

Huaqian Yang (China)

Farah Yasmin (United States)

Yang Yu (China)

Qiuhuan Yuan (China)

Naufal S. Zagidullin (Russia)

Luca Zazzeron (United States)

Ying Zhang (China)

Dongying Zhang (China)

Li Zhang (China)

Cui Zhang (China)

Donghui Zhang (China)

Wei Zhao (China)

Zhiwei Zhao (China)

Yue Zhao (China)

Tao Peter Zhong (China)

Zhan Zhong-Qun (China)

Manhong Zhou (China)

Xiaodong Zhou (China)

Haining Zhou (China)

Zihua Zhou (China)

Weizhong Zhu (China)

Ni Zhu (China)

